# HOPS/Tmub1 involvement in the NF-kB-mediated inflammatory response through the modulation of TRAF6

**DOI:** 10.1038/s41419-020-03086-5

**Published:** 2020-10-15

**Authors:** Marina Maria Bellet, Stefania Pieroni, Marilena Castelli, Danilo Piobbico, Francesca Fallarino, Luigina Romani, Maria Agnese Della-Fazia, Giuseppe Servillo

**Affiliations:** Department of Experimental Medicine, University of Perugia, 06132 Perugia, Italy

**Keywords:** Stress signalling, Epigenetics in immune cells

## Abstract

HOPS/Tmub1 is a ubiquitously expressed transmembrane ubiquitin-like protein that shuttles between nucleus and cytoplasm during cell cycle progression. HOPS causes cell cycle arrest in G_0_/G_1_ phase, an event associated to stabilization of p19^Arf^, an important tumor suppressor protein. Moreover, HOPS plays an important role in driving centrosomal assembly and maintenance, mitotic spindle proper organization, and ultimately a correct cell division. Recently, HOPS has been described as an important regulator of p53, which acts as modifier, stabilizing p53 half-life and playing a key role in p53 mediating apoptosis after DNA damage. NF-κB is a transcription factor with a central role in many cellular events, including inflammation and apoptosis. Our experiments demonstrate that the transcriptional activity of the p65/RelA NF-κB subunit is regulated by HOPS. Importantly, *Hops*^*−/−*^ cells have remarkable alterations of pro-inflammatory responses. Specifically, we found that HOPS enhances NF-κB activation leading to increase transcription of inflammatory mediators, through the reduction of IκBα stability. Notably, this effect is mediated by a direct HOPS binding to the E3 ubiquitin ligase TRAF6, which lessens TRAF6 stability ultimately leading increased IKK complex activation. These findings uncover a previously unidentified function of HOPS/Tmub1 as a novel modulator of TRAF6, regulating inflammatory responses driven by activation of the NF-κB signaling pathway. The comprehension on how HOPS/Tmub1 takes part to the inflammatory processes in vivo and whether this function is important in the control of proliferation and tumorigenesis could establish the basis for the development of novel pharmacological strategies.

## Introduction

Hepatocyte Odd Protein Shuttling (HOPS), also named TMUB1 (Transmembrane and ubiquitin-like protein-1) (from herein after HOPS) is a highly conserved and ubiquitous protein encoded by a gene isolated from a cDNA library of rat regenerating liver^1^, together with other 40 novel genes, some of which have been characterized during the recent years^2–4^. *Hops* gene transcribes a single mRNA which is translated in three different proteins. HOPS is characterized by an ubiquitin-like (UBL) domain and three trans-membrane leucine-rich regions^5^. Indeed, a proteomic analysis performed on liver and blood cells identified HOPS as a nuclear envelope transmembrane protein^6^. Furthermore, a classical Nuclear Export Signal (NES) domain drives the export outside the nucleus through the exportin chromosome region maintenance 1 protein (CRM-1)^1,7^.

In quiescent hepatocytes, HOPS is mainly localized in the nucleus. The export of HOPS outside the nucleus of proliferating cells during liver regeneration appears to be mediated by epidermal growth factor (EGF) and cyclic adenosine monophosphate (cAMP)^1^. Instead, IL-6 could be responsible of the increased expression of HOPS during liver regeneration, possibly through a mechanism involving the binding of the transcription factor C/EBPβ to the promoter of HOPS and the activation of its transcription^8,9^. HOPS has a role in mitotic spindle assembly and centrosome duplication and is involved in the control of cell cycle progression^10–12^. Moreover, it was found to participate in the ERAD pathway, where it plays an important role in the control of sterol-accelerated ubiquitination and degradation of the cholesterol biosynthetic enzyme HMG-CoA reductase^13^. HOPS has been shown to interact with the tumor suppressors p19^Arf^ enabling its stabilization through the involvement of the nucleolar protein nucleophosmin (NPM)^14^. Recently, HOPS has been reported to be a fundamental regulator of mitochondrial p53 activity during DNA damage-induced apoptosis^15^. HOPS is abundantly expressed in the brain, where it plays a role in the regulation of basal synaptic transmission^16,17^.

NF-κB/Rel is a family of inducible transcription factors playing a pivotal role in inflammatory and immune responses^18,19^. There are five family members in mammals: RelA (p65), RelB, c-Rel, NF-κB1 (p105/p50), and NF-κB2 (p100/p52) and different NF-κB complexes are formed by homo- and hetero-dimerization between family members. In most unstimulated cell types, NF-κB complexes are retained in the cytoplasm by a family of inhibitory proteins known as inhibitors of NF-κB (IκBs). Activation of NF-κB by agents, such as lipopolysaccharide (LPS), typically involves the phosphorylation on IκB by IκB kinase (IKK) complex, which in turn results in IκB degradation and NF-κB release^20^. The genes regulated by NF-κB include those controlling programmed cell death, cell adhesion, proliferation, innate and adaptive immune responses and tissue remodeling^21^.

Because HOPS was previously isolated from immune cells^11^, and is known to be induced by the pro-inflammatory cytokine IL-6^9^, in this study we explored the possibility that HOPS might affect the pro-inflammatory responses. Our findings, comparing MEFs and immune cells derived from *Hops* wild type (*Hops*^*+/+*^) and knock-out (*Hops*^*−/−*^) mice, strongly suggested that HOPS contributes to the normal activation of the pro-inflammatory response by identifying a novel mechanism in modulating the NF-κB signaling pathway.

## Results

### Reduced inflammatory response in *Hops*^*−*/*−*^ cells

**Fig. 1:**
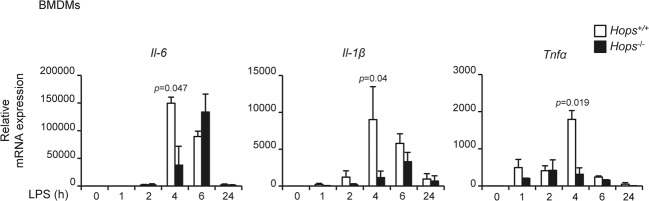
Absence of HOPS affects pro-inflammatory gene transcription. *Hops*^*−*/*−*^ cells are less responsive to LPS stimulation. Time course of mRNA expression of *Il-6, Il-1β*, and *Tnfα* after LPS stimulation (10 µg/ml) for the indicated times (hours, h) in *Hops*^+/+^ and *Hops*^*−*/*−*^ bone marrow derived macrophages (BMDMs), measured by qPCR. Values are normalized to *18*S housekeeping gene. Graphs show fold changes in gene expression compared with unstimulated cells. All the values are the mean ± SD (*n* = 3 mice). Statistically significant changes are shown.

To explore whether HOPS could modulate the inflammatory response, we used cultured primary cells as bone marrow derived macrophages (BMDMs), splenocytes and mouse embryonic fibroblasts (MEFs) isolated from *Hops*^+/+^ and *Hops*^*−/−*^ mice comparing their expression profile of pro-inflammatory genes. At first, we followed the timing of expression of various cytokines after LPS stimulation. We observed that the expression of the pro-inflammatory genes *Il-6*, *Il-1β*, and *Tnfα* was significantly reduced in BMDMs from *Hops*^*−*/*−*^ compared to *Hops*^+/+^ mice exclusively 4 hours after LPS activation (Fig. 1). Similarly, the expression of pro-inflammatory cytokines and enzymes (*Il-6*, *Il-1β, Cox2*) was reduced in splenocytes and MEFs derived from *Hops*^*−*/*−*^ compared to *Hops*^+/+^ mice, although at a lower level compared to BMDMs (Supplementary Fig S1).

However, HOPS impact in pro-inflammatory response is not related to the modulation of NF-κB subunits, whose amount seems unaltered upon HOPS over or down-expression (Supplementary Fig S2).

### HOPS modulates NF-κB activation

**Fig. 2:**
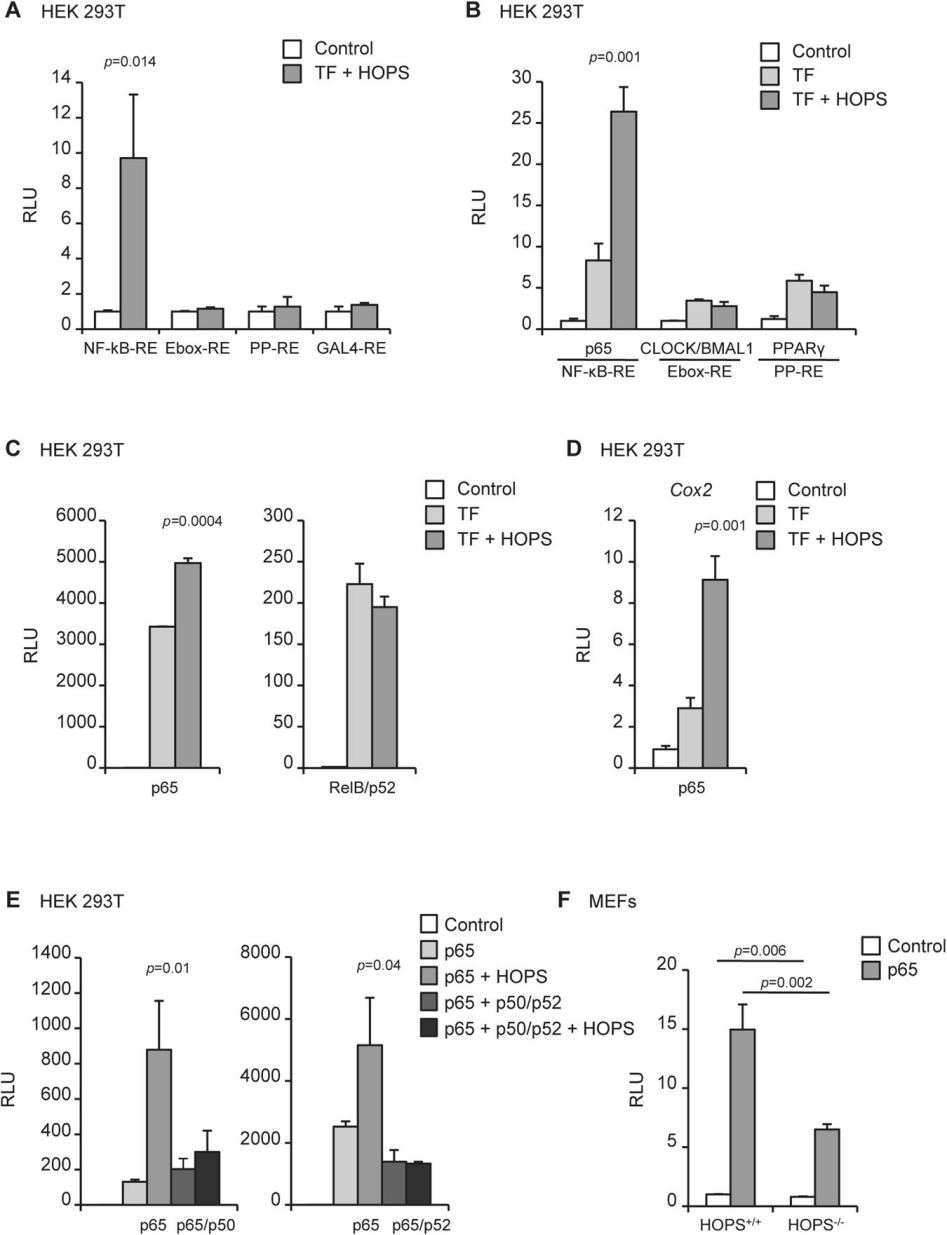
Overexpression of HOPS enhances NF-κB-driven transcription. **A** Transcriptional activity of HOPS. myc-tagged HOPS was cotransfected in HEK-293T cells with constructs containing multiple copies of the responsive elements (RE) NF-κB-RE, E-box-RE, PP-RE, GAL4-RE. After normalization for transfection efficiency using β-galactosidase activity, reporter gene activity was expressed as Relative Luciferase Units (RLU). Activity in control vector transfected cells was set to 1. All the values are the mean ± SD (*n* = 3). Statistically significant changes are shown. **B** Effect of HOPS on NF-κB-dependent transcription in HEK-293T. Same as in **A**, except that vectors expressing the transcription factors (TF) p65, CLOCK/BMAL1 or PPARγ were cotransfected with constructs containing NF-κB-RE, E-box-RE, or PP-RE, respectively, with or without myc-HOPS. **C** Effect of HOPS on p65 or RelB/p52-mediated transcription in HEK-293T. Same as in **A**, except that vectors expressing p65 or RelB and p52 were cotransfected with constructs containing NF-κB-RE, with or without myc-HOPS. **D** Effect of HOPS on NF-κB-induced transactivation of *Cox2*-promoter in HEK-293T. Same as in **B**, except that the vector expressing p65 was cotransfected with a construct containing *Cox2* full promoter, with or without myc-HOPS. **E** Effect of HOPS on transactivation of NF-κB-REs induced by p65 or p65/p50 and p65/p52 dimers in HEK-293T. Same as in **C**, except that vector expressing p65 was transfected alone or cotransfected with p50 (left) or p52 (right), together with NF-κB-RE, with or without myc-HOPS. **F** NF-κB activation is HOPS-dependent in MEFs. p65 was cotransfected with NF-κB-RE in *Hops*^+/+^ and *Hops*^*−*/*−*^ cells.

The NF-κB transcription factor plays a central role in inflammatory response induced by LPS. We sought to explore the possibility that HOPS could be functionally relevant in modulating NF-κB-mediated transcriptional activation. To address this question, we performed a luciferase-based assay on transiently transfected HEK 293T cells, by using plasmids in which the luciferase gene expression was under the control of a promoter region containing a specific responsive element (RE). Interestingly, HOPS overexpression resulted in increased transactivation of NF-κB-RE, while no difference was observed in the transactivation of other transcription factors (TF)-binding sites, including E-box-RE, PP-RE, and GAL4-RE, thus suggesting a specificity of action of HOPS on NF-κB-RE (Fig. 2A).

Because HOPS does not act as transcription factor, we hypothesized a role of HOPS in modulating the activity of TFs acting on NF-κB-RE. To explore this assumption, a luciferase assay was conducted by transfecting HEK 293T cells with NF-κB-REs, together with the p65 subunit of NF-κB, in presence or absence of HOPS. At the same time, other REs were similarly transfected and activated by their own specific TFs, in particular the E-box-REs were activated by the circadian factors CLOCK/BMAL1, and PP-REs by co-transfecting the nuclear receptor PPARγ. The result showed that HOPS significantly enhanced the transactivation ability of the p65 subunit of NF-κB, whereas no differences in the activation of the other analyzed REs were observed in the presence of HOPS (Fig. 2B).

Different heterodimers drive the canonical (p65) and the non-canonical (RelB/p52) pathways of NF-κB, involved in different and specific immune functions^22^. Based on this assumption, we explored whether the effect of HOPS was specific for the p65 subunit of NF-κB or instead could be observed also after activation by RelB/p52. The result showed that HOPS overexpression in HEK 293T specifically increased the p65-induced transactivation, while no effect was observed on RelB/p52 subunits (Fig. 2C).

The increased transactivation induced by p65 in the presence of HOPS on isolated NF-κB-RE suggests that HOPS might exert the same function on a full promoter, where REs for other TFs are present together with NF-κB-RE, and take parts in the modulation of gene expression in different functional moments. Thus, we measured the luciferase activation induced by HOPS on the promoter of a classical NF-κB target gene, *Cox-2*. Similarly to the effect observed on isolated NF-κB-REs, HOPS overexpression determined a significant 3-folds increase in p65-mediated induction of the luciferase transcription driven by *Cox-2* promoter (Fig. 2D).

p65 is the transcriptionally active subunit of NF-κB, which needs to homo- or hetero-dimerize in order to activate transcription. Since the most common dimers acting during the activation of the canonical pathway of NF-κB are the heterodimers p65/p50 and, on less extent, p65/p52, we asked whether the enhancement in transcription produced by HOPS could affect the activity of the dimers. Surprisingly, we observed that while HOPS was able to enhance the transcription induced by p65 alone, no additional effect was observed during overexpression of p65 together with p50 or p52 (Fig. 2E), suggesting a specific role of HOPS on the p65 subunit of NF-κB, which is not exerted when engaging p50 or p52.

Finally, to demonstrate that lack of HOPS affects NF-κB-mediated transcription, we performed a luciferase assay in mouse embryonic fibroblasts (MEFs) derived from *Hops*^*−/−*^ mice and *Hops*^+/+^ littermates. We observed that the NF-κB-RE basal activation was significantly reduced in *Hops*^*−/−*^ compared to *Hops*^*+/+*^ MEFs, with a consistent decrease in luciferase activity after RelA transfection in MEFs *Hops*^*−/−*^ respect to *Hops*^+/+^(Fig. 2F).

Altogether these results suggest a functional role of HOPS as a potential modulator of NF-κB transcriptional activity.

### HOPS-mediated induction of NF-κB target genes transcription

**Fig. 3:**
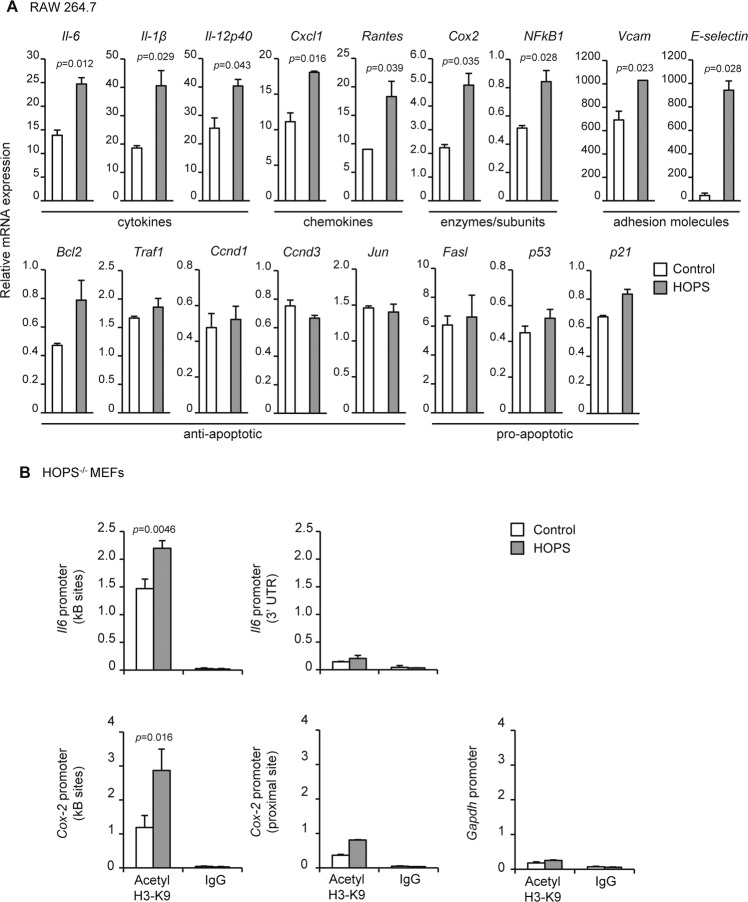
HOPS potentiates p65 target genes transcription. **A** mRNA expression of NF-κB-target genes in RAW 264.7 cells transfected with p65 with or without HOPS, measured by qPCR. Values in untransfected cells (not shown) are set to 1. All values are the mean ± SD (*n* = 3). Statistically significant changes are shown. **B** ChIP from *Hops*^*−*/*−*^ MEFs cells transfected with myc-HOPS or an empty vector. Cells were collected after 24 h of transfection, subjected to dual cross-link and ChIP with acetyl-H3(K9) antibody or rabbit IgG was performed. Primers for NF-κB sites and 3′ UTR of *Il-6* promoter and NF-κB sites and proximal regions of *Cox2* promoter were used for qPCR. NF-κB sites binding was detected within *Gapdh* promoter as negative control. All the values are the mean ± SD (*n* = 3). Statistically significant changes are shown.

To further confirm the role of HOPS on p65-mediated transcriptional activity, we decided to explore the effect of HOPS overexpression on endogenous transcription of NF-κB target genes. RAW 264.7 cells were transfected with p65 alone or in combination with HOPS. Total RNA was extracted and levels of expression of different NF-κB target genes were analyzed by qPCR. We observed that mRNA expression of the pro-inflammatory cytokines *Il-6, Il-1β*, and *Il-12p40*, the chemokines *Cxcl1* and *Rantes*, the inducible enzyme *Cox2*, the NF-κB subunit p50 (*NF-κB1*), and the adhesion molecules *Vcam* and *E-selectin*, were induced to higher levels in presence of HOPS (Fig. 3A), while other cytokines such as *Ifn-β* and *Il-10* and the NF-κB inhibitor *I-κBα* were not significantly increased (Supplementary Fig S3). None of the NF-κB anti-apoptotic (*Bcl2*, *Traf1*, *Ccnd1*, *Ccnd3*, *Jun*) or pro-apoptotic (*Fasl, p53, p21*) target genes were upregulated in HOPS overexpressing cells (Fig. 3A), thus indicating a specificity for target genes directly involved in the pro-inflammatory response.

Next, we sought to determine whether this effect was associated to changes at the chromatin level at target genes promoter. A classical marker of a transcriptionally active chromatin state is the acetylation of lysine 9 and 14 of histone H3 (H3 K9/K14) at specific sites of the promoter of the gene of interest. Therefore, we performed chromatin immunoprecipitation (ChIP) by using a specific anti-acetyl H3 histone antibody or IgG as a control. In HOPS expressing cells, the result showed an increased acetylation of H3 at K9 on *Il-6* promoter, specifically at the NF-κB binding sites containing region, but not in the 3′ UTR region of the same promoter (Fig. 3B). Similarly, acetylation was significantly increased at the *Cox2* promoter NF-κB binding regions, but not at its proximal regions (Fig. 3B). In accordance with these results, we observed reduced trimethylation of H3 K9, a marker of chromatin silencing, in HOPS overexpressing cells (Supplementary Fig S4). Importantly, we did not observe a direct binding of HOPS to NF-κB responsive elements in the same experimental conditions, thus indicating that HOPS has not a transcriptional control, but takes place upstream in the NF-κB activation pathway (Supplementary Fig S4). No specific signal was detected in the *Gapdh* promoter used as a negative control (Fig. 3B).

All together, these results suggest an effect of HOPS in the regulation of NF-κB target genes transcription, which is associated to enhanced histone acetylation and reduced histone methylation at NF-κB binding sites.

### HOPS shuttles between the nucleus and the cytoplasm during LPS treatment

**Fig. 4:**
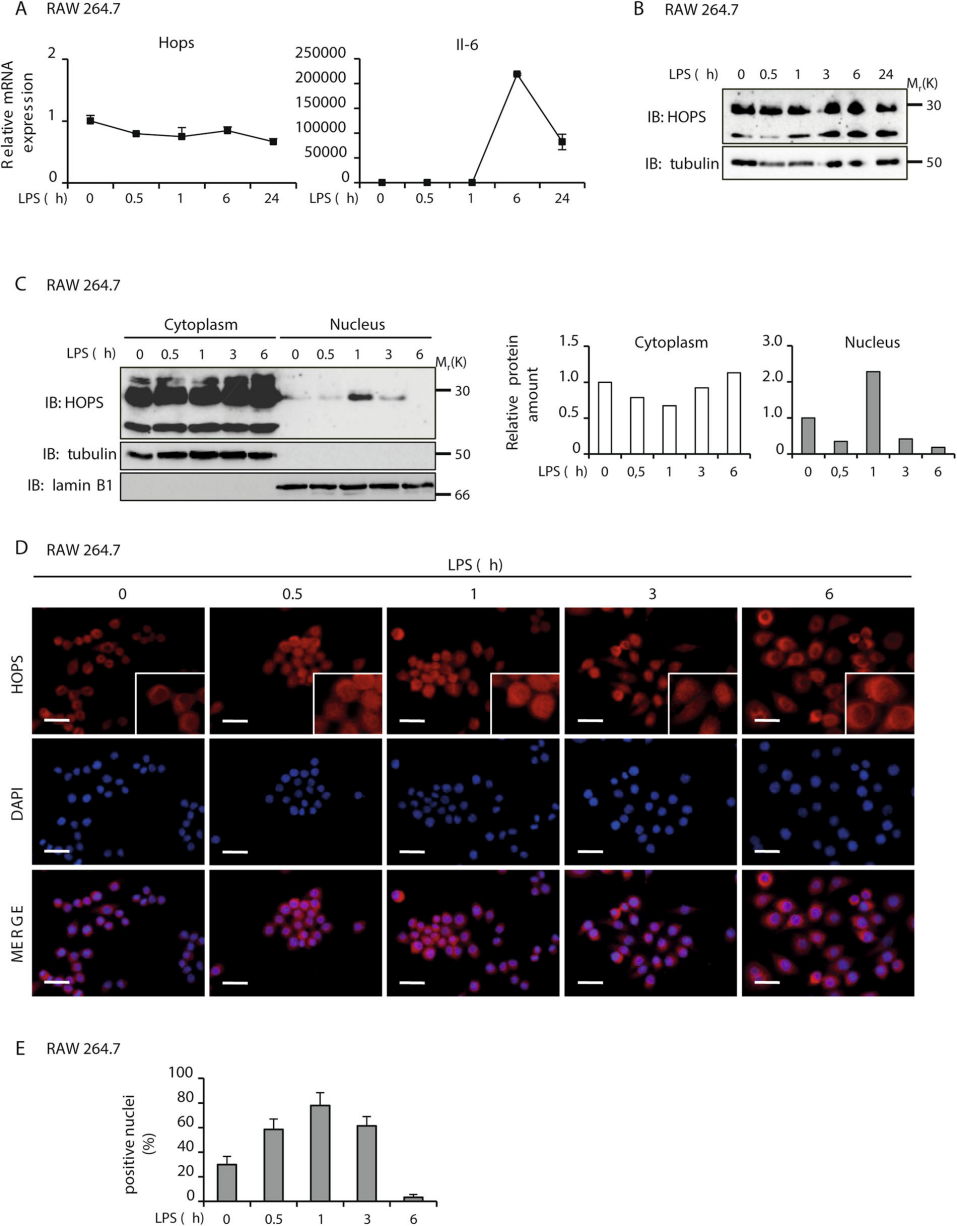
HOPS shuttles between the nucleus and the cytoplasm during LPS treatment. **A** mRNA expression of *Hops* and *Il-6* in RAW 264.7 cells treated with LPS for the indicated time, measured by qPCR. All the values are the mean ± SD (*n* = 3). **B** Protein expression of HOPS in RAW 264.7 cells treated with LPS for the indicated time. α-tubulin was used as loading control. **C** Nuclear and cytoplasmic extracts prepared from RAW 264.7 cells treated with LPS for the indicated times. WB analysis was performed using anti-HOPS antibody. α-tubulin and lamin B1 were used as loading control for cytoplasm and nuclei, respectively. Graphs on the right show densitometric analysis for HOPS expression levels in cytoplasmic and nuclear fractions. **D** HOPS immunolocalization in RAW 264.7 cells treated with LPS for the indicated times, analyzed using HOPS antibody (red). Nuclei were DAPI stained (blue). Representative images are shown. Bars, 10 μm. **E** Percentage of HOPS-positive nuclei observed and counted at the indicated times after LPS treatment. Bars represent mean ± SD (*n* = 10 field of observation form at least 3 independent samples).

Because HOPS influences NF-κB activity during an inflammatory stimulus, we asked whether, its own expression might change in the same conditions. To explore this possibility, we measured both mRNA and protein levels of HOPS after LPS treatment. No differences were observed in mRNA expression levels of *Hops* between samples harvested at different time of treatment, while *Il-6* mRNA expression was quantified as a control of the efficacy of the treatment (Fig. 4A). Similarly, no change in HOPS protein level was observed in the same condition (Fig. 4B).

As HOPS, similarly to NF-κB, is a nucleus-cytoplasm shuttling protein in dependence of stress stimuli, we analyzed whether its cellular localization changes after stimulation with LPS. After a time-course with LPS, RAW264.7 cells were harvested and nuclear and cytoplasmic extracts were used for a WB analysis with anti-HOPS antibody. In untreated cells, HOPS was prevalently distributed in the cytoplasm (Fig. 4C). Interestingly, 1 hour after LPS administration, HOPS appears to migrate to the nucleus reducing its amount in the cytoplasm, moving back rapidly to the cytoplasm at 3 and 6 hours from treatment (Fig. 4C).

The HOPS nucleus-cytoplasmic shuttling was further confirmed through immunofluorescence studies by using anti-HOPS antibody (Fig. 4D and Supplementary Fig. S5). In particular, HOPS localization changed from cytoplasmic to nuclear at 1 hour following LPS treatment, when the number of HOPS-positive nuclei significantly increased (Fig. 4E). Then HOPS shuttled back to the cytoplasm at 6 hours of treatment (Fig. 4D and Supplementary Fig. S5). In conclusion, these results suggest an active shuttling of HOPS between nucleus and cytoplasm in cells activated by an inflammatory stimulus that is associated to its role in the modulation of NF-κB activity.

### HOPS induces activation of the p65/RelA subunit of NF-κB

**Fig. 5:**
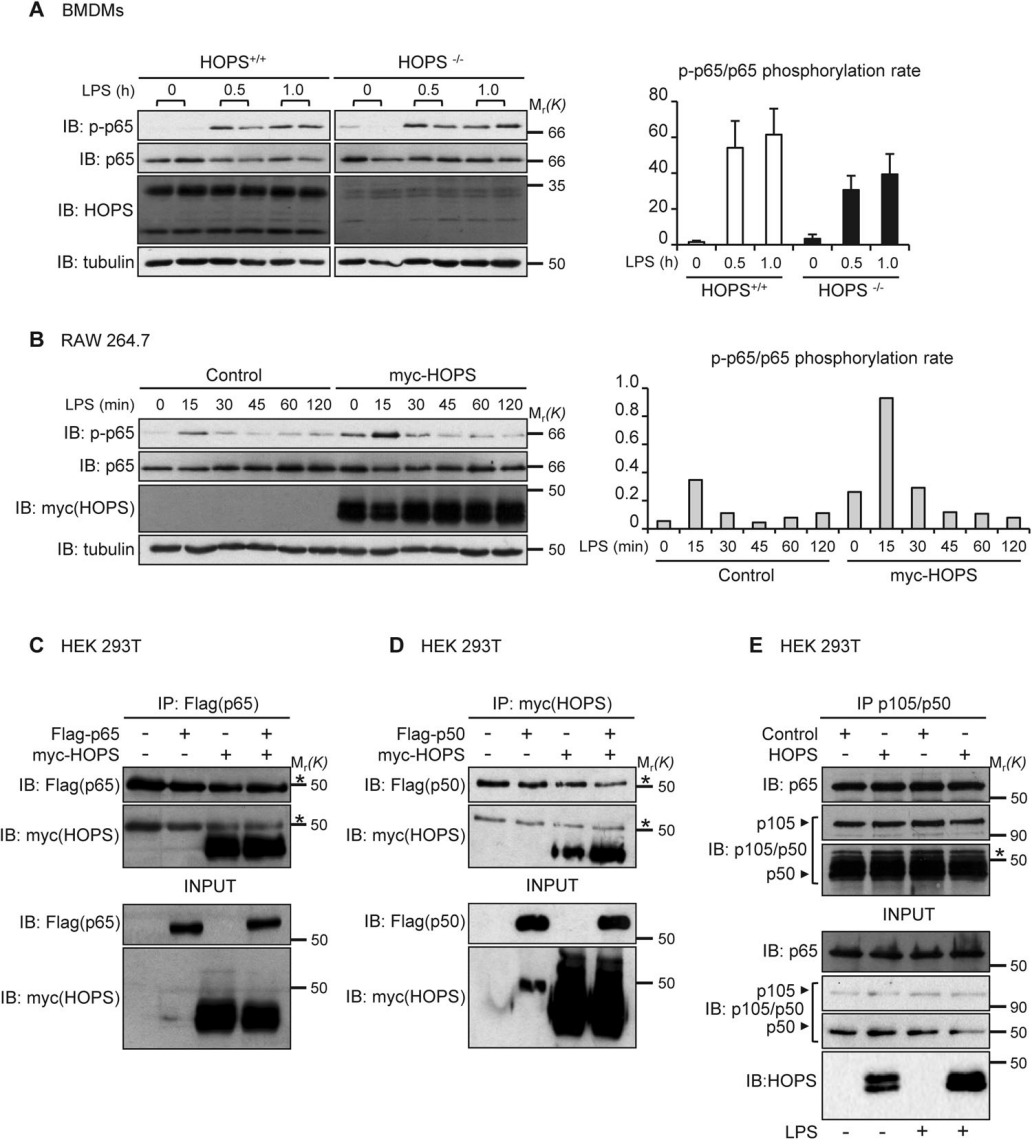
HOPS increases p65 phosphorylation. **A** Expression of p-p65 (Ser536) and total p65 in BMDMs from *Hops*^+/+^ and *Hops*^*−*/*−*^ mice, after LPS treatment. Anti-HOPS antibody was used as control of HOPS expression. α-tubulin was used as loading control. Diagram on the right shows densitometric analysis for p-p65 levels relative to total p65 amount. **B** Expression of p-p65 (Ser536) and total p65 in RAW 264.7 cells transfected or not with myc-HOPS and treated with LPS for the indicated times. Anti-myc antibody was used as HOPS transfection control. α-tubulin was used as loading control. Densitometric analysis for p-p65 phosphorylation rate versus total p65 expression levels is shown on the right. **C** HEK 293 T cells were cotransfected with myc-HOPS and Flag-p65. HOPS was immunoprecipitated with anti-myc antibody, and co-immunoprecipitated proteins were revealed with anti-Flag and anti-myc antibodies. Total cell lysate input was revealed with anti-Flag and anti-myc antibodies. **D** Same as in **C**, except that HEK 293T cells were cotransfected with myc-HOPS and Flag-p50. **E** HEK 293 T cells were transfected with HOPS or an empty vector (Control) and treated or not with LPS (10 μg/ml). Endogenous p65/p50 binding was studied by protein immunoprecipitation with anti-p105/p50 antibody and WB analysis with anti-p65 antibody. Total cell lysate input was revealed with anti-p65, anti-p105/p50 and anti-HOPS antibodies. Asterisks in **C**, **D**, **E** indicate IgG. Arrows in **E** indicates p105 and p50 specific bands.

We then addressed the question whether the increased NF-κB-mediated transcription induced in the presence of HOPS could be related to increased activation of p65. NF-κB-activating agents can induce phosphorylation of p65 at multiple sites. To evaluate this possibility, we explored whether HOPS could affect p65 phosphorylation at Serine 536 (Ser536) induced by LPS treatment. The experiments were performed in BMDMs derived from *Hops*^*+/+*^ and *Hops*^*−/−*^ mice. WB analysis, by using a specific anti-p-p65 (Ser536) antibody, revealed that, p65 level of phosphorylation was reduced, displaying a defective activation of NF-κB in cells lacking HOPS (Fig. 5A). p65 phosphorylation and subsequent activation following LPS treatment was also analyzed by transfecting myc-HOPS in RAW 264.7 cells. WB analysis revealed increased phosphorylation rate in cells overexpressing HOPS compared to those transfected with control vector (Fig. 5B).

To establish whether the effect induced by HOPS depends on a direct binding between HOPS and p65, a co-immunoprecipitation (co-IP) experiment was carried out by ectopically overexpressing myc-HOPS and Flag-p65 fusion proteins in HEK 293T cells. WB performed using anti-Flag and anti-myc antibodies did not reveal binding between HOPS and p65 (Fig. 5C). Similarly, no binding was observed between HOPS and the NF-κB subunit p50 (Fig. 5D). Because the absence in binding obtained by co-IP of the single subunits and HOPS, we asked whether p65/p50 binding was influenced by the presence of HOPS. A co-IP experiment was performed by immunoprecipitating p105/p50 in cells overexpressing myc-HOPS or an empty vector, and WB revelation with anti-p65 antibody showed that HOPS did not interfere with the binding between the two proteins (Fig. 5E).

### HOPS controls IκBα stability

As previously described, the NF-κB signaling pathway is characterized by steps of phosphorylation, ubiquitination and degradation of proteins involved in the cascade of activation^23^. Phosphorylation, ubiquitination, and proteasomal degradation of the inhibitor of NF-κB, IκBα, is a central event after LPS stimulation.

**Fig. 6:**
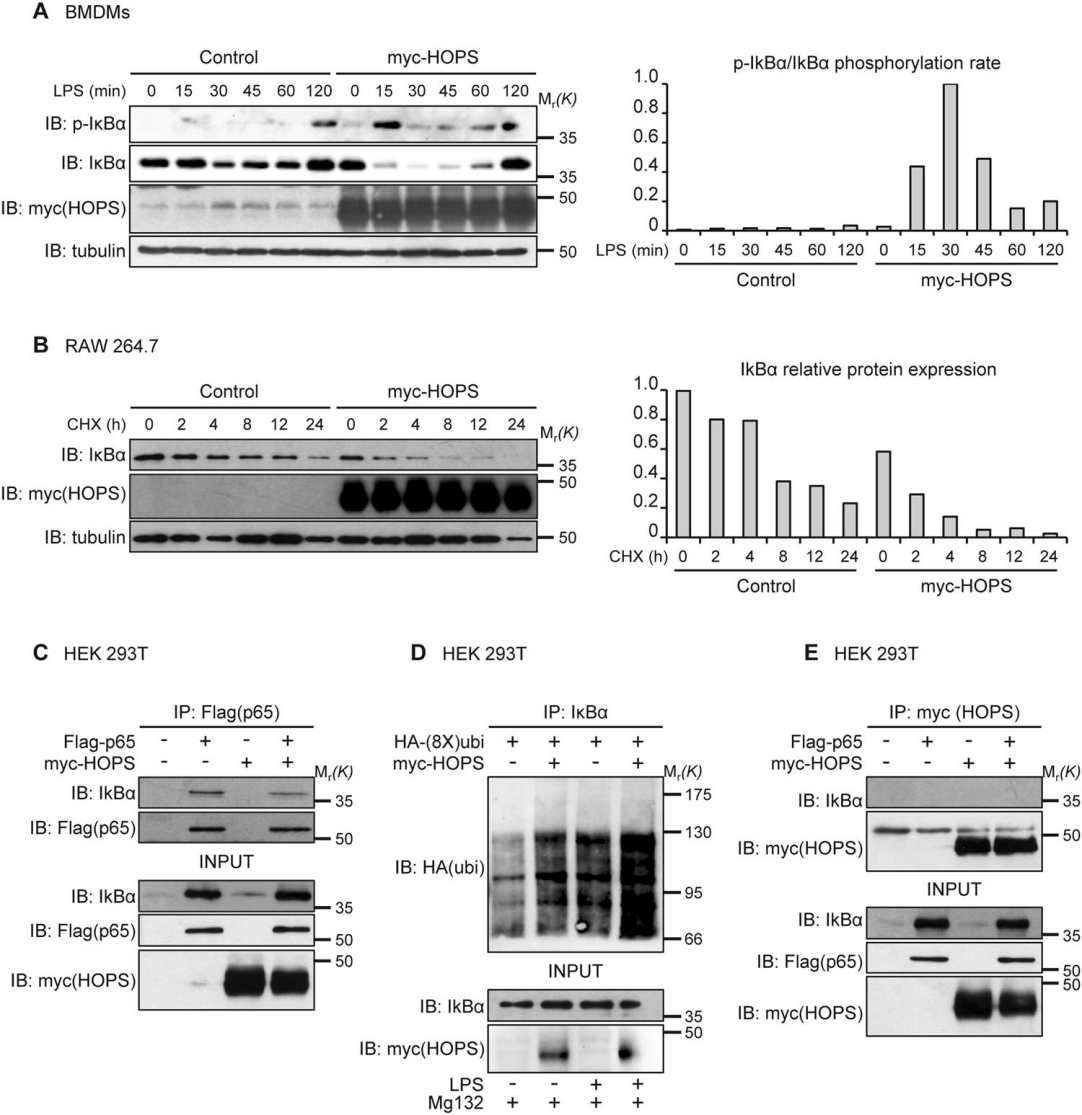
HOPS interferes with IκBα stability in the absence of direct binding. **A** Expression of p-IκBα (Ser32) and total IκBα in RAW 264.7 cells transfected with or without myc-HOPS and treated with LPS for the indicated times. Anti-myc antibody was used as control of transfection. α-tubulin was used as loading control. Densitometric analysis for p-IκBα phosphorylation rate versus total IκBα expression levels is shown on the right. **B** Expression of IκBα in RAW 264.7 cells transfected with or without myc-HOPS and treated with CHX (100 μM) for the indicated times. α-tubulin was used as loading control. Densitometry for IκBα expression levels is shown on the right. **C** HEK 293T cells were cotransfected with myc-HOPS and Flag-p65. p65 was immunoprecipitated with anti-Flag antibody, and coimmunoprecipitated proteins were revealed by WB analysis with anti-IκBα anti-Flag antibodies. Total cell lysate input was revealed with anti-Flag, anti-IκBα and anti-myc antibodies. **D** HEK 293T cells were transfected with HA-(8X)ubiquitin and myc-HOPS or empty vector. 24 h post-transfection cells were exposed to MG132 (10 μM) for 6 h. 30 min before lysis, cells were treated with LPS (10 μg/ml) or left untreated. Anti-IκBα immunoprecipitations were immunoblotted with anti-HA to reveal the amount of ubiquitin-bound IκBα. IκBα and HOPS (anti-myc) were probed in total cell lysates as experimental input (**E**) HEK 293T cells were cotransfected with myc-HOPS and Flag-p65. HOPS was immunoprecipitated with anti-myc antibody, and coimmunoprecipitated proteins were revealed by WB analysis with anti-IκBα and anti-myc antibodies. Total cell lysate input was revealed with anti-Flag, anti-IκBα and anti-myc antibodies.

Because the results obtained showed a difference in p65 phosphorylation in cells overexpressing HOPS, we monitored IκBα phosphorylation following LPS stimulation in cells overexpressing HOPS compared to control cells. As reported^24^, LPS induces rapid phosphorylation at 15 minutes and subsequent proteolysis of IκBα in control cells, an event necessary for the activation of NF-κB. In cells overexpressing HOPS, the phosphorylation of IκBα was strongly increased and IκBα degradation was significantly enhanced, in agreement with the role of HOPS in the activation of p65 (Fig. 6A).

Based on these results, we wondered whether HOPS modulation of the NF-κB pathway, at the IκBα level, could be associated to its ubiquitin-like protein function. For this reason, we hypothesized that IκBα stability might change in the presence of HOPS. After transfection of RAW 264.7 cells with HOPS or a control vector, cells were treated with cycloheximide (CHX). WB analysis of protein lysates from these cells using anti-IκBα antibody revealed a clear reduction of IκBα stability in the presence of HOPS (Fig. 6B).

IκBα binding to the p65/RelA subunit is responsible of its cytoplasmic retention in unstimulated cells. The reduced stability of IκBα in the presence of HOPS could lead to reduced binding to, and consequently increased activation of p65. To test this hypothesis, an anti-Flag antibody was used to immunoprecipitate Flag-p65 in cells overexpressing or not HOPS. As expected, IκBα binding to p65 appear to be weakly reduced by the presence of HOPS (Fig. 6C). Of note, experiments carried on in HOPS naïve or overexpressed cells with no modulation on either p65 or IκBα levels showed an unaltered p65/IκBα binding, demonstrating that HOPS effect in controlling NF-κB pathway does not reside in the active control of p65/IκBα interaction, but rather in the modulation of IκBα levels (Supplementary Fig S6).

Because the role of HOPS as a ubiquitin-like domain containing protein, we wondered whether it could be involved in ubiquitin-mediated degradation of IκBα occurring following inflammatory stimuli such as LPS, a crucial event in the activation of the NF-κB signaling pathway. We performed experiments by transfecting myc-HOPS together with ubiquitin in RAW 264.7 cells stimulated or not with LPS for 30 minutes. Treatment with the proteasome inhibitor MG132 leads to accumulation of ubiquitin-conjugated proteins (Fig. 6D). Results showed that the presence of HOPS increased the fraction of IκBα bound to ubiquitin in both untreated cells and, more importantly, after LPS stimulation. However, this event was not mediated by a direct binding of HOPS to IκBα. Indeed, absence of any specific binding between HOPS and IκBα was revealed by an immunoprecipitation experiment in cells transfected with myc-HOPS, Flag-p65 or both (Fig. 6E).

In conclusion, these results corroborate the idea of HOPS control in NF-κB signaling pathway revealing an indirect control in p65 and IκBα activation. Of note, HOPS doesn’t bind both proteins suggesting an upstream control occurring during pro-inflammatory cascade.

### HOPS interacts with TRAF6 ubiquitin ligase and modulates TRAF6 degradation and NF-κB pathway activation

**Fig. 7:**
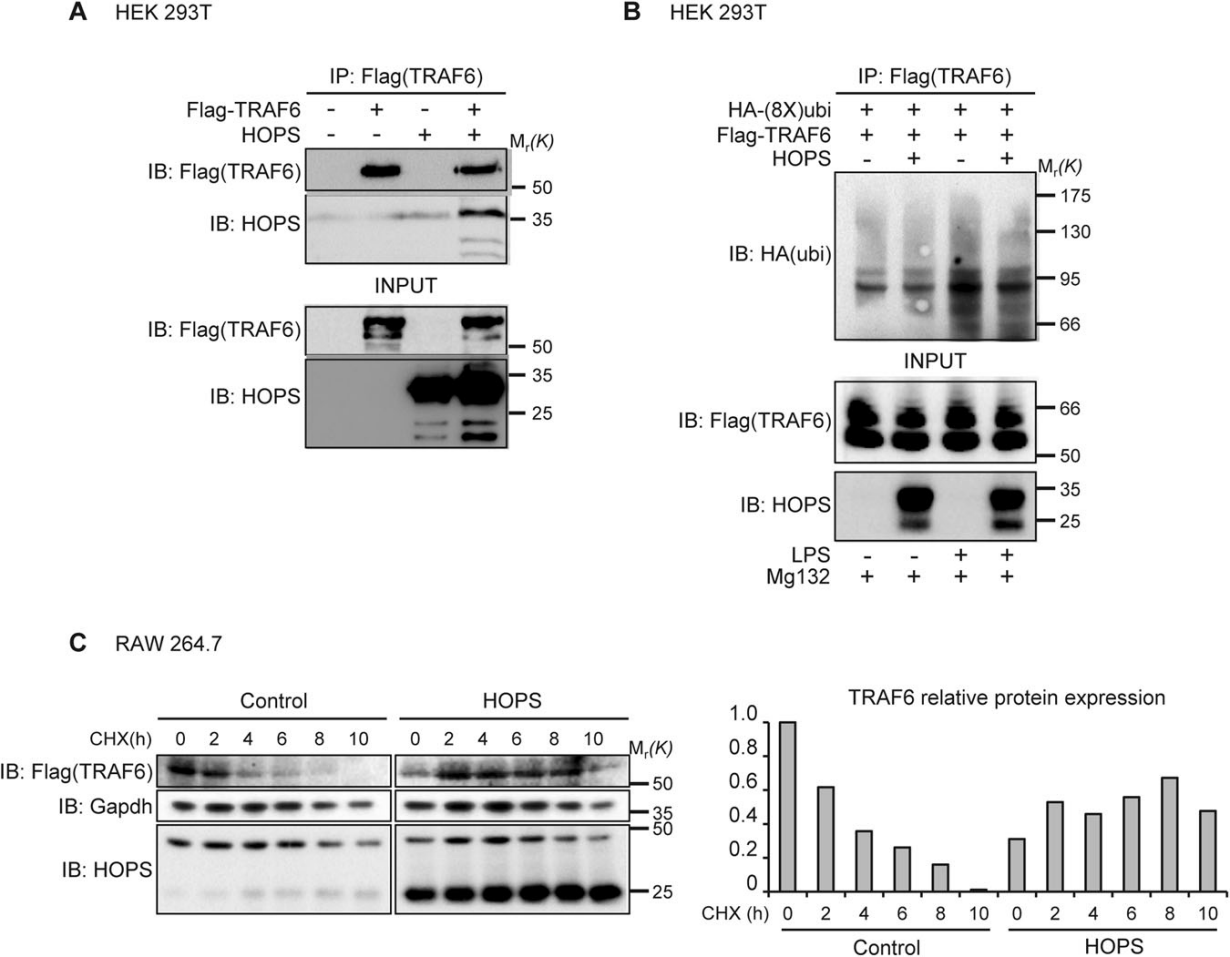
HOPS interacts with TRAF6 and controls its stability. **A** HEK 293T cells were cotransfected with HOPS and Flag-TRAF6. HOPS was immunoprecipitated with anti-HOPS antibody, and immunoprecipitated proteins were detected by WB analysis using anti-Flag and anti-HOPS antibodies. Total cell lysate input was revealed with anti-Flag and anti-HOPS antibodies. **B** HEK 293T cells were cotransfected with vectors encoding for Flag-TRAF6 and HA-(8X)ubiquitin in the presence of HOPS or not. 24 h post-transfection cells were exposed to MG132 (10 μM) for 4 h. 30 min before lysis, cells were treated with LPS (10 μg/ml) or left untreated. Anti-Flag (for TRAF6) immunoprecipitations were immunoblotted with anti-HA to reveal the amount of ubiquitin-bound TRAF6. Total cell lysate input was revealed with anti-Flag and anti-HOPS antibodies. **C** Expression of TRAF6 in RAW 264.7 cells transfected with or without HOPS and treated with CHX (100 μM) for the indicated times. Gapdh was used as loading control. Densitometry for TRAF6 expression levels is shown on the right.

In the NF-κB canonical pathway, phosphorylation, inducing the subsequent ubiquitination of IκBα, is carried out by IKK kinase complex, composed of two catalytic subunits, IKKα and IKKβ, and one essential regulatory subunit, IKKγ (NEMO). Upon Toll-like receptor (TLR) activation by LPS, a RING-domain ubiquitin ligase, TNF-α Receptor Activating Factor 6 (TRAF6) promotes the polyubiquitination of several targets, including NEMO. These events of poly and autoubiquitination mediated by TRAF6 are required for signaling to downstream kinases. Indeed, Lys-63 linked polyubiquitin chain acts as scaffold to assemble protein kinase complexes and mediate their activation^25,26^. Because HOPS does not bind directly p65 and IκBα, but regulates their activation, we assessed whether HOPS intervention on NF-κB canonical cascade activation could origin from a direct TRAF6 interaction. At first, we explored a possible binding between HOPS and TRAF6. We performed co-immunoprecipitation assay with myc-HOPS and Flag-TRAF6 transfected cells. Notably, the co-IP revealed a direct binding between the two proteins (Fig. 7A). HOPS/TRAF6 interaction was furtherly confirmed by co-IP assay in naïve cells expressing endogenous levels of both the proteins involved (Supplementary Fig S7). To verify whether such interaction allows HOPS to control TRAF6 ubiquitin-dependent degradation, we carried out an in vivo ubiquitination assay upon LPS induction and MG132 proteasomal inhibition. We transfected Flag-TRAF6, together with HOPS and HA-(8X)ubiquitin in HEK 293T. Results showed that HOPS reduced the rate of TRAF6 ubiquitinated species in LPS-stimulated cells (Fig. 7B). The reduced ubiquitination of TRAF6 in the presence of HOPS is mediated by a physical interaction between HOPS and TRAF6. TRAF6 half-life spans from 2 to 4 hours depending on cell type. To further demonstrate that TRAF6 stabilization is related to HOPS, we inhibited protein *de novo* synthesis by CHX treatments in wild type and HOPS over expressing RAW 264.7 cells. As expected increased HOPS levels lengthened TRAF6 half-life (Fig. 7C).

Then, we evaluated the HOPS-mediated TRAF6 stabilization by analyzing the resulting NEMO activation, after LPS induction^27^. After transfection of HEK 293T cells with HA-(mono)ubiquitin and Flag-TRAF6 with or without HOPS and relative controls, we observed that HOPS overexpression increased NEMO polyubiquitination (Supplementary Fig S7). Thus, as a direct effect of TRAF6 stabilization, the ubiquitin-based activation was observed for IKKγ, supporting the hypothesis of the HOPS-related NF-κB cascade activation.

**Fig. 8:**
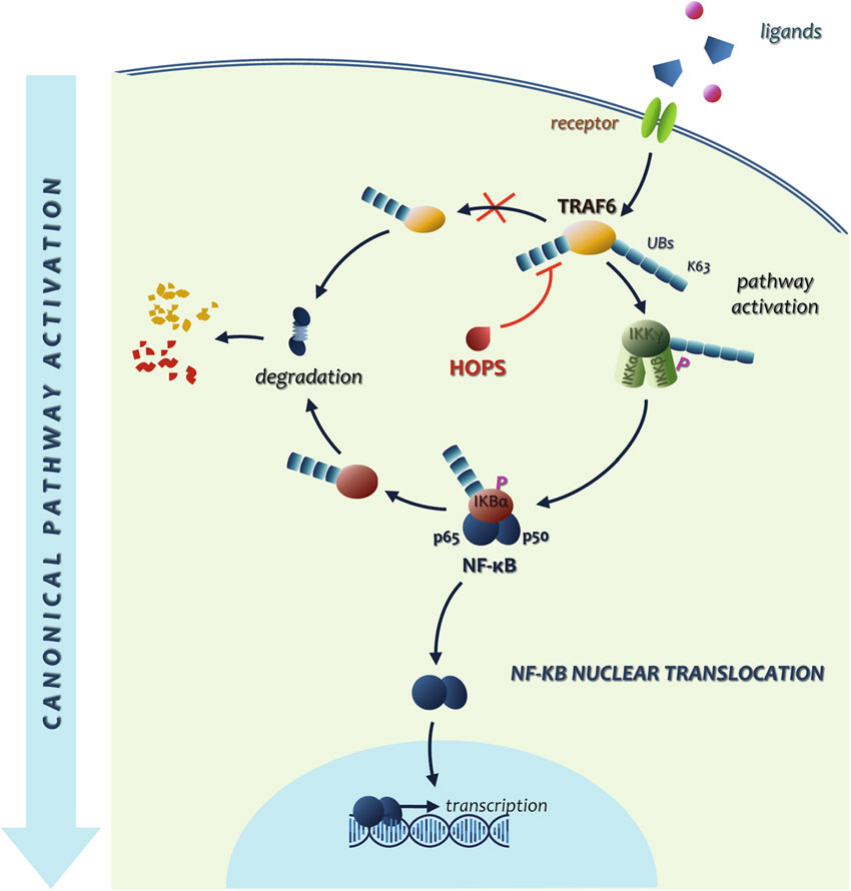
Schematic representation of HOPS activity in the canonical pathway activation. HOPS inhibits TRAF6 proteasomal degradation leading to a higher TRAF6 protein amount in the cell. TRAF6 triggers IKKγ, which in turn activates NF-κB with subsequent nuclear translocation. Once in the nucleus NF-κB acts as transcription factor and regulates its target genes transcription.

All together these data reveal a potential mechanism through which the ubiquitin-like domain containing protein HOPS modulates the NF-κB signaling pathway, by interacting with, and modulating TRAF6 ubiquitin ligase polyubiquitination, thus resulting in promotion of the NF-κB-driven pro-inflammatory response (Fig. 8).

## Discussion

The identification and characterization of novel genes involved in the regulation of important biological processes, such as cell division, differentiation, inflammation and oncogenesis, is one of the possible approaches to improve the understanding of the complex molecular mechanisms regulating different cellular functions. The complete comprehension of how these events are regulated is crucial for the identification of those defects responsible for the development of many diseases, from inflammatory diseases to cancer. Our previous reports showed that the protein HOPS plays a central role in different cellular functions, including maintenance of genomic stability, correct proliferation and progression through the cell cycle^1,10,14,28,29^.

Here we found that HOPS is also important in ensuring a proper inflammatory response. The analysis of *Hops* knockout cells revealed a defective activation of macrophages and other cell types following an inflammatory stimulus such as LPS. A large number of genes encoding for pro-inflammatory factors, including macrophages-produced cytokines and chemokines, are direct NF-κB target genes. The ability of HOPS in modulating NF-κB activation and function was revealed by a series of in vitro experiments in HOPS overexpressing cells, confirming that HOPS has a relevant role in supporting the transcription of NF-κB target genes.

Notably, our results show that no specific control of a protein modifier such as HOPS has a direct binding with NF-κB subunits p65 or p50 or IκBα, supporting the possibility that the regulation occurs upstream in the NF-κB signaling.

Indeed, we established that HOPS acts as ubiquitin-like protein with a number of proteins, modifying the stability of important oncosuppressors such as p19^Arf^ and p53. The intricate control in NF-κB pathway open the question about at what level HOPS could play its role in controlling in the NF-κB activation. Several studies have described the sumoylation control in NF-κB pathway controlling IκB or IKK, while our results seem to show a lack of binding with these effectors indicating a HOPS control in proteins regulating upstream NF-κB pathway cascade^28,29^.

This hypothesis was confirmed by experiments that demonstrated that HOPS interacts with the ubiquitin ligase TRAF6 and modulates its ubiquitination leading, in turn, to the NF-κB canonical pathway control.

Because HOPS has been described as a shuttling protein, moving from the nucleus to the cytoplasm following different stress stimuli, this evidence led to the hypothesis that HOPS could change localization to perform its function of strengthen the activity of NF-κB. The experiments performed after inflammatory stimulus revealing an active shuttling of HOPS between the nucleus and the cytoplasm, let us suppose an increase in TRAF6 stability and subsequent activation of the downstream signaling pathways. These data indicate a direct control in TRAF6 activation by a modifier such HOPS (Fig. 8). A normally functioning HOPS appears to be necessary to obtain an efficient inflammatory response, and its absence affects the normal activation of immune and non-immune cells following an inflammatory stimulus such as LPS. In vivo experiments will be crucial to understand whether the reduced inflammatory response observed in *Hops*^*−/−*^ cells lead to a different susceptibility to infections in these mice. Moreover, once defined the role of HOPS in the inflammatory process, it will be important to integrate it into the broader landscape of cell proliferation and tumor development, in which HOPS carries out its known functions. The comprehension of the relationship between HOPS and NF-κB, and the role of HOPS in the inflammatory process, will help in the definition of the role of HOPS in the proliferative process and during carcinogenesis.

## Methods

### Plasmids

RelA cFlag pcDNA3 (Addgene plasmid # 20012)^30^, RelB cFlag pcDNA3 (Addgene plasmid # 20017), p50 cFlag pcDNA3 (Addgene plasmid # 20018) and p52 cFlag pcDNA3 (Addgene plasmid # 20019) were a gift from Stephen Smale. Plasmids expressing both β-galactosidase for transfection control (LacZ pCMV) and luciferase (luc) for luminometry based expression (pLV-5X-NF-κB Luc, pGL3-3X-Ebox Luc, PPRE-3X-TK Luc, pGL2-5X-Gal4 Luc, pGL3basic-Cox2 Promo Luc) were described previously^31–35^. HOPS encoding sequence was amplified and cloned in pCS2+MT and pSG5 as previously described^1^. N-terminal myc-tagged plasmids myc-CLOCK-pSG5, Flag-myc-BMAL1 pCS2+MT, Flag-PPARγ1 were previously described^34–36^. pMT123 (HA-(8X)ubiquitin) and pRK5-HA-Ubiquitin (Addgene plasmid # 17608) were gifts from Dirk Bohmann and Ted Dawson respectively. Flag-TRAF6-WT (Addgene plasmid #21624) was a gift from John Kyriakis^37^.

### Reagents and antibodies

Ultrapure lipopolysaccharide (LPS), MG132 and Cycloheximide (CHX) were purchased from Sigma-Aldrich (St. Louis, Missouri, USA). Antibodies against phospho-p65 (Ser536), phospho-IκBα (Ser32), p65, p105/p50, and anti-HA were from Cell Signaling Technology (Danvers, Massachusetts, USA) anti-α-tubulin, anti-Gapdh, anti-myc and anti-Flag M2 were from Sigma-Aldrich, anti-IκBα, was from Santa Cruz Biotechnology (Santa Cruz, California, USA), anti-acetyl-histone H3 (acetyl K9) from Millipore (Burlington, Massachusetts, USA) anti-trimethyl-histone H3 (trimethyl K9) and anti-lamin B1 from Abcam (Cambridge, UK) anti-TRAF6 was from Thermo Fisher Scientific (Waltham, Massachusetts, USA). The rabbit polyclonal antibody used for the detection of HOPS was previously described^1^.

### Cell culture

HEK 293T cells (Human Embryonic Kidney 293T, ATCC, Manassas, Virginia, USA) and MEFs form *Hops*^−/−^ were maintained in DMEM (Euroclone, Milan, Italy) supplemented with 10% fetal bovine serum (FBS), antibiotics, L-glutamine and not essential aminoacids (NEAA) (EuroClone) and cultured at 37°C in 5% CO_2_. RAW 264.7 cells (mouse leukemic monocyte macrophage cell line, ATCC) were grown in RPMI1640 (EuroClone) supplemented with 10% FBS, antibiotics, L-glutamine, Hepes and β-mercaptoethanol (EuroClone), and cultured at 37°C in 5% CO_2_. MEFs, splenocytes and BMDMs were generated from *Hops*^+/+^ or *Hops*^−/−^ C57BL/6 homozygous sibling mice and the preparation protocol was adapted from previous^38–40^.

### Transient transfection, luciferase assay and cells treatments

Cells were seeded in 24-well plates at a density of 7.5 × 10^4^ cells per well. Cells were transfected with FUGENE®6 (Promega, Madison, Wisconsin, USA) according to the manufacturer’s protocol. Cell extracts were subjected to a luminometry-based luciferase assay, and luciferase activity was normalized by β-galactosidase activity. For TRAF6 half-life cells at 80% confluence were treated with CHX (100 μM) for the time points indicated and analyzed by WB.

### Western blotting (WB) and immunoprecipitation (IP)

WB experiments were performed as previously described^41^. For the immunoprecipitation assay, cells were harvested 24 h after transfection, washed twice with cold phosphate buffered saline (PBS, Sigma-Aldrich) and lysed in RIPA buffer (Tris/HCl pH 8.0, 50 mM, NaCl 150 mM, SDS 0.1%, sodium deoxycholate 1%, Triton X-100 1%), supplemented with protease inhibitor cocktail (PIC) and phenylmethylsulfonyl fluoride (PMSF) (Sigma-Aldrich). Lysates were sonicated 2 × 10 sec and cleared, and proteins were quantified by Bio-Rad Protein assay (Bio-Rad, Hercules, California, USA). Lysates were incubated overnight at 4°C with the specific antibody, followed by 2 h incubation with protein G- or protein A-Sepharose beads (GE Healthcare Lifescience, Little Chalfont, UK). Immune complexes were washed four times in washing buffer and boiled in 2× sample buffer. The proteins were separated by electrophoresis on SDS-PAGE and detected using specific antibodies. Protein band intensities were quantified by densitometric analysis using Image J software.

### In vivo ubiquitination assay

Cells were transfected with pMT123 (HA-(8X)ubiquitin), pRK5-HA-Ubiquitin, Flag-TRAF6 and HOPS-pSG5. Cells were treated or not with LPS for 30 min (10 µg/ml) and MG132 (100 µM) for 6 h. Cells were then lysed in RIPA buffer with PIC and PMSF and then sonicated, clarified and processed as for IP. The ubiquitinated species were analyzed by western blotting with anti-HA specific antibody.

### Nuclear and cytoplasmic fractionation

Cells were seeded in 6-well plates at a density of 2 × 10^5^ cells per well. After LPS treatment, cells were harvested in ice-cold PBS, washed twice and lysed with hypotonic buffer (Hepes-KOH pH 7.9, MgCl2, KCl, DTT) supplemented with PIC and PMSF and 0,5% of Nonidet P-40 (NP40, Sigma-Aldrich). Following a brief centrifugation at 4°C, the supernatant was recovered as cytoplasmic fraction. The pellet (nuclear fraction) was washed twice with hypotonic buffer and lysed in Laemmli buffer.

### Immunofluorescence

Cells were fixed in 4% paraformaldehyde, permeabilized in 0.2% Triton X-100, and incubated in blocking buffer (BSA). The specific primary antibody to HOPS, used for immunostaining, was added to the blocking buffer and left in incubation overnight. For detection, anti-rabbit Alexa Fluor 555-conjugate (Thermo Fisher Scientific) was used as the secondary antibody. Nuclei were stained with 4,6-diamidino-2-phenylindole (DAPI, Sigma-Aldrich). Images were examined using a Zeiss Axioplan fluorescence microscope controlled by Spot-2 cooled camera (Diagnostic Instruments Inc., Michigan, USA).

### RNA extraction and quantitative real-time PCR (qPCR)

For gene expression analysis by qPCR, total RNA was extracted with TRIzol Reagent (Thermo Fisher Scientific) and processed according to the manufacturer instructions. 1 μg of RNA from each sample was retrotranscribed using iScript RT Supermix (Bio-Rad Laboratories), and 4-fold diluted cDNA was used for each PCR reaction. For a 20 μl of PCR, 50 ng of cDNA was mixed with primers to final concentration of 150 nM and 10 μl of Brilliant SYBR®Green QPCR Master Mix and ROX (Agilent Technologies) as reference dye. The reaction was first incubated at 95°C for 3 min, followed by 40 cycles at 95°C for 30 sec and 60°C for 1 min. qPCR was performed by monitoring in real-time the increase in fluorescence on an Mx3000P™Real-Time PCR detector system (Agilent Technologies Inc., Santa Clara, California, USA). Results were analyzed using the 2-DDCt method to calculate the relative level of each mRNA and expressed as a ratio relative to *18S* rRNA housekeeping gene. Bars represent the mean ± SD (*n* = 3–4). Primers sequences are available upon request.

### Chromatin Immunoprecipitation (ChIP) assay

Dual crosslinking ChIP assay was used^42^. Briefly, cells were washed three times with room temperature PBS and PBS with 1 mM MgCl2 was added. Disuccinimidyl Glutarate (DSG, Sigma-Aldrich) was added to a final concentration of 2 mM for crosslinking and incubated 45 min at RT, formaldehyde (Sigma-Aldrich) was added to a final concentration of 1% (v/v) and cells incubated for 15 min for dual crosslinking. Glycine was added to a final concentration of 0.1 M and incubated for 10 min to quench formaldehyde cross-linking. After harvesting, cells were lysed in 500 μl ice-cold cell lysis buffer (50 mM Tris/HCl pH 8.0, 85 mM KCl, 0.5% NP40, 1 mM PMSF, protease inhibitor cocktail) (Sigma-Aldrich) for 10 min on ice. Nuclei were precipitated by centrifugation (3000×*g* for 5 min), resuspended in 600 µl ice-cold RIPA buffer (50 mM Tris/HCl pH 8.0, 150 mM NaCl, 1 mM EDTA pH 8.0, 1% Triton X-100, 0.1% SDS, 0.1% sodium deoxycolate, 1 mM PMSF, protease inhibitor cocktail) (Sigma-Aldrich) and incubated on ice for 30 min. Sonication was performed to obtain DNA fragments 100–600 bp in length using Soniprep 150 (MSE Sanyo, London, UK).

### Animals

Mice were used exclusively to prepare primary cells cultures. The study was carried on in accordance with ethical principles of the latest version of the Declaration of Helsinki. All animal experiments were carried out in accordance with the U.K. Animals (Scientific Procedures) Act, 1986 and associated guidelines (EU Directive 2010/63/EU for animal experiments). All experiments involving animals were done according to the guidelines of the University of Perugia Ethical Committee and the European Communities Council Directive 2010/63/EU.

### Statistical analysis

Statistical analysis was performed routinely with at least three biological repeats unless otherwise specified. Statistical significance of variations between test groups was assessed by two-tailed Student’s *t*-tests. Experimental data were analyzed to calculate *p* values using the Excell software, with *p* values <0.05 considered as statistically significant. Experimental data were illustrated as mean ± SD.

## Supplementary information

Supplementary FIg.S1

Supplementary FIg.S2

Supplementary FIg.S3

Supplementary FIg.S4

Supplementary FIg.S5

Supplementary FIg.S6

Supplementary FIg.S7

Supplementary FIg.S8

Supplementary Figure Legends
