# Supplementary figures and images for: HOPS/Tmub1 involvement in the NF-kB-mediated inflammatory response through the modulation of TRAF6
Source: Cell Death Dis. 2020 Oct 15;11(10):865. doi: 10.1038/s41419-020-03086-5 (PMC7567074; doi:10.1038/s41419-020-03086-5)

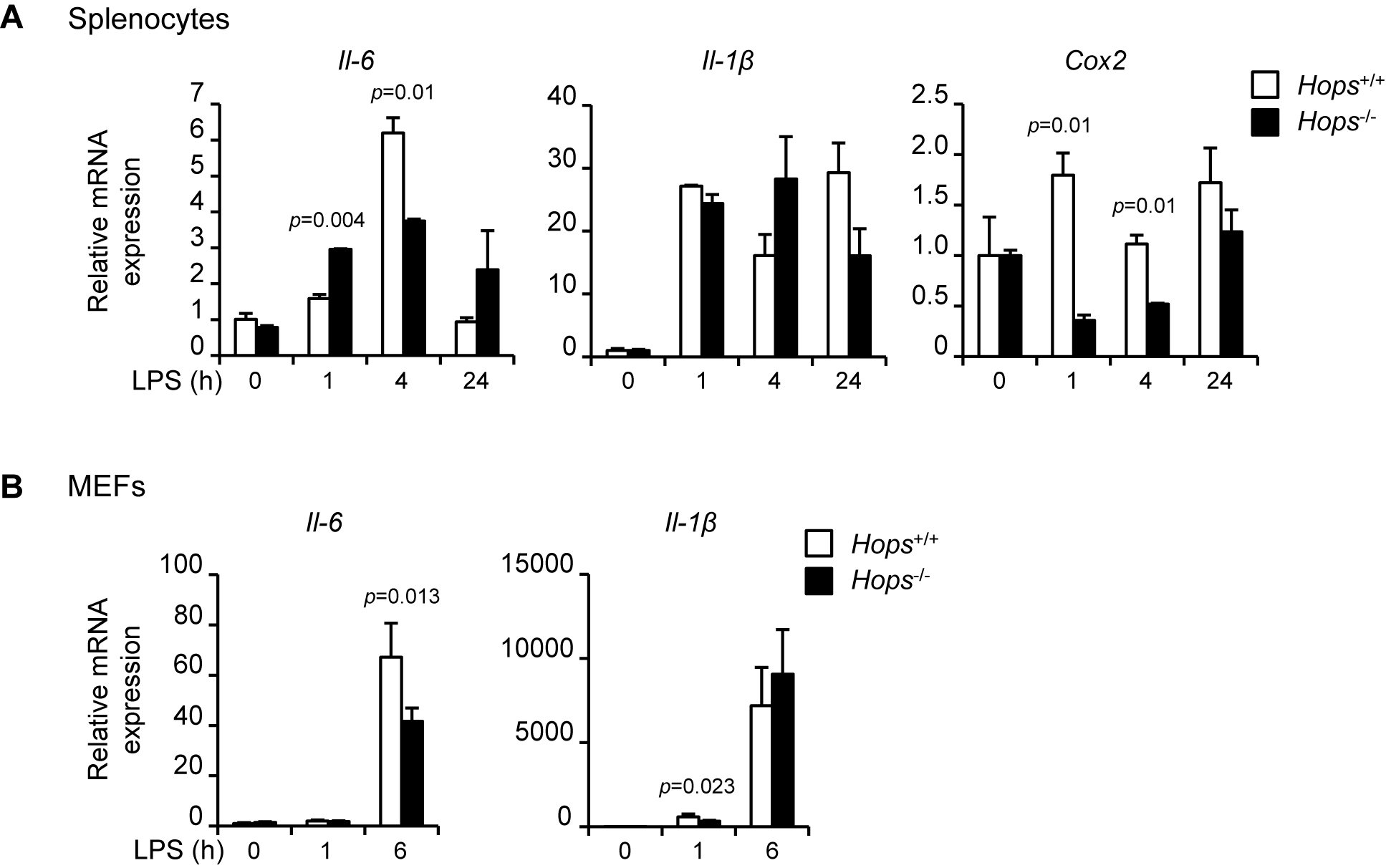

Supplement: Supplementary file 1 — Supplementary FIg.S1 [file 41419_2020_3086_MOESM1_ESM.tif]

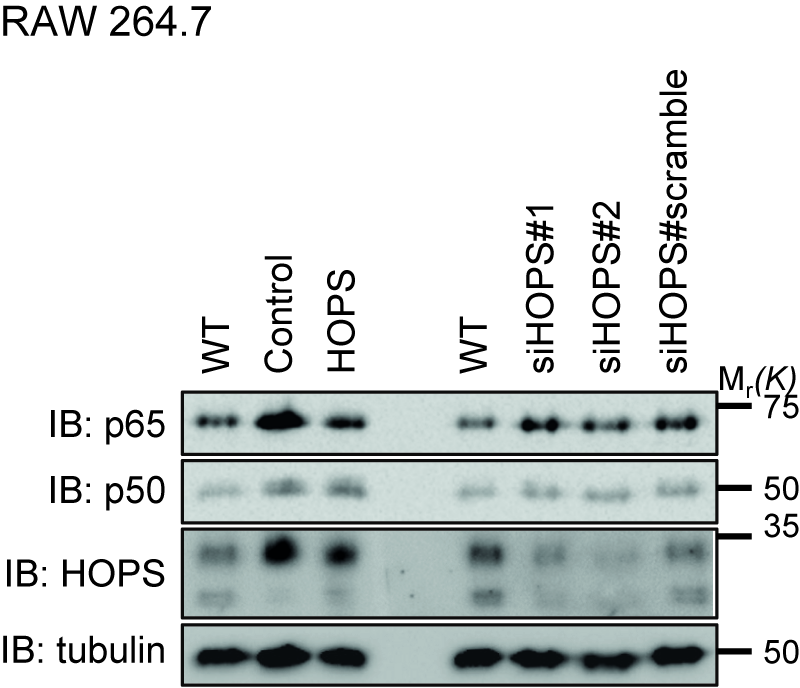

Supplement: Supplementary file 2 — Supplementary FIg.S2 [file 41419_2020_3086_MOESM2_ESM.tif]

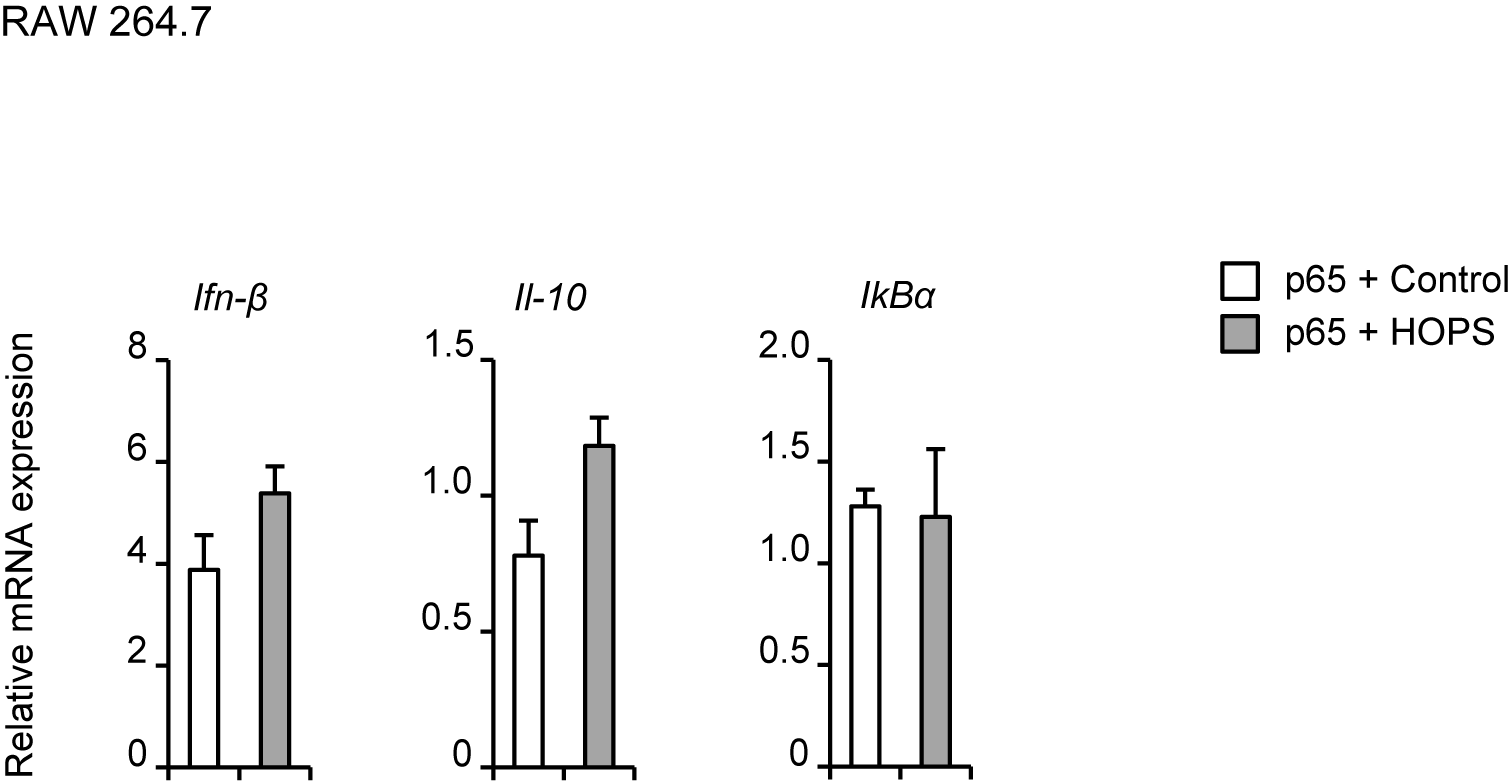

Supplement: Supplementary file 3 — Supplementary FIg.S3 [file 41419_2020_3086_MOESM3_ESM.tif]

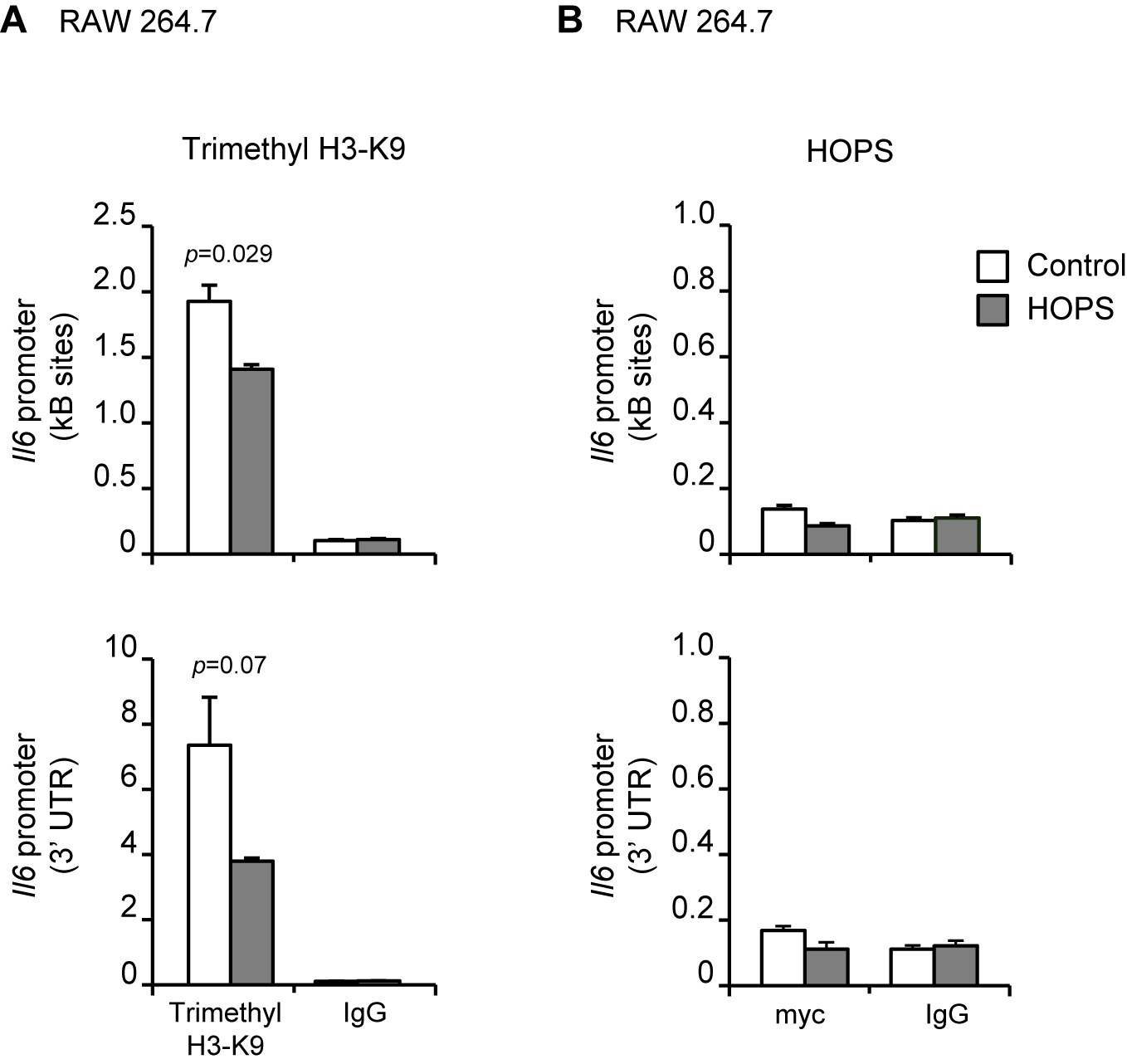

Supplement: Supplementary file 4 — Supplementary FIg.S4 [file 41419_2020_3086_MOESM4_ESM.tif]

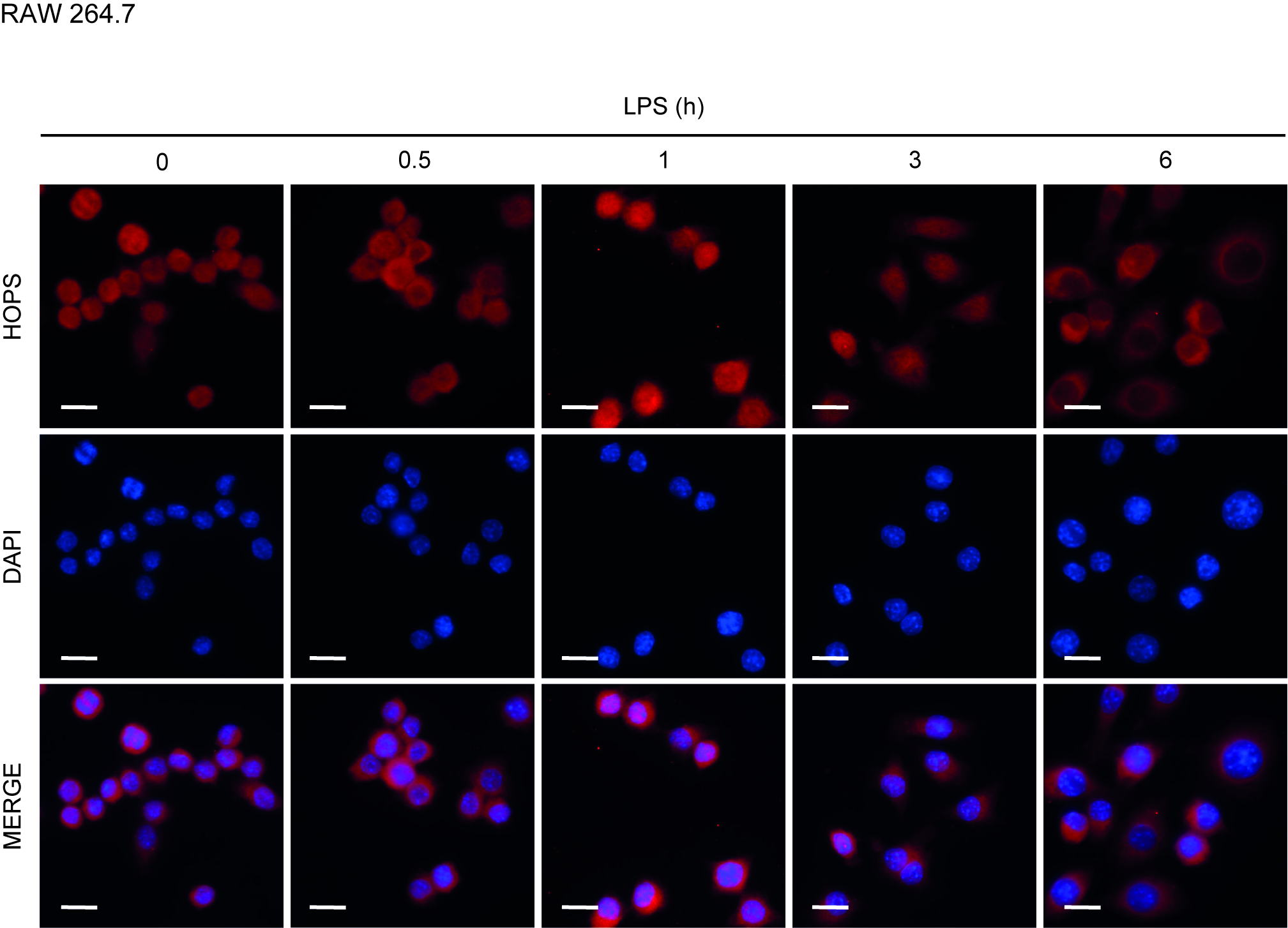

Supplement: Supplementary file 5 — Supplementary FIg.S5 [file 41419_2020_3086_MOESM5_ESM.tif]

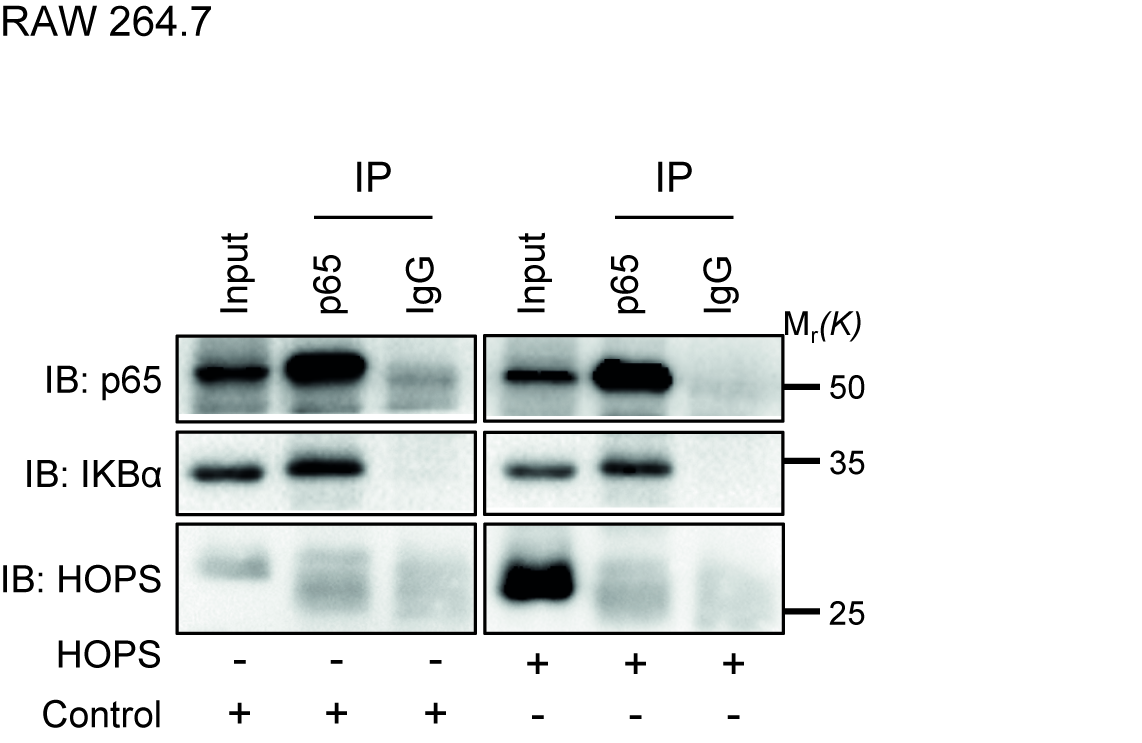

Supplement: Supplementary file 6 — Supplementary FIg.S6 [file 41419_2020_3086_MOESM6_ESM.tif]

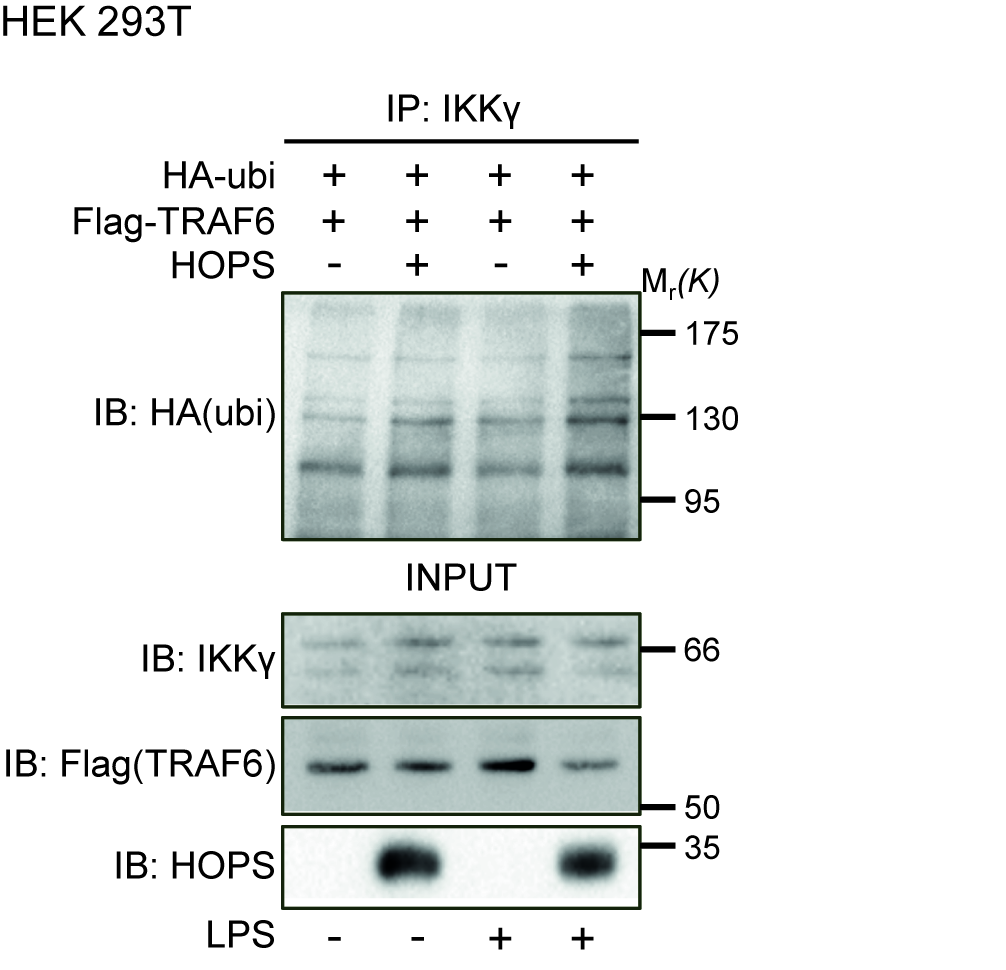

Supplement: Supplementary file 7 — Supplementary FIg.S7 [file 41419_2020_3086_MOESM7_ESM.tif]

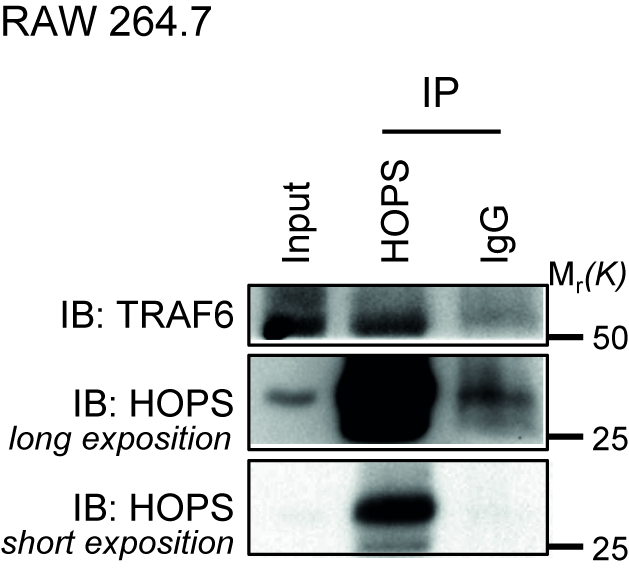

Supplement: Supplementary file 8 — Supplementary FIg.S8 [file 41419_2020_3086_MOESM8_ESM.tif]
