# Supplementary material for: HOPS/Tmub1 involvement in the NF-kB-mediated inflammatory response through the modulation of TRAF6
Source: Cell Death Dis. 2020 Oct 15;11(10):865. doi: 10.1038/s41419-020-03086-5 (PMC7567074; doi:10.1038/s41419-020-03086-5)
Supplement: Supplementary file 9 — Supplementary Figure Legends [file 41419_2020_3086_MOESM9_ESM.docx]

**Supplementary Fig.S1. Responsiveness to LPS stimulation**. mRNA expression of different cytokines after LPS stimulation (10µg/ml) in *Hops*^+/+^ and *Hops*^-/-^ splenocytes (**A**) and MEFs (**B**), measured by qPCR. Graphs show fold changes in gene expression. Values are the mean ± SD (n=3 mice). Statistically significant changes are shown.

**Supplementary Fig.S2. Analysis of NF-κB p65 and p50 protein expression relative to HOPS protein amount in the cell**. Data showed no significant modification of p65/p50 protein amount in both HOPS overexpressing or silenced RAW 264.7 cell line. Lysates were normalized to tubulin. Representative image is shown.

**Supplementary Fig.S3. Expression of NF-κB-target genes**. RAW264.7 cells were transfected with p65 together or not with HOPS, mRNA amount was measured by qPCR. The values are relative to those of *18S*-mRNA levels. Value of untransfected cells (not shown) are set to 1. All the values are the mean ±SD (n=3).

**Supplementary Fig.S4. ChIP-assay on HOPS transfected RAW264.7 cells. A** ChIP was performed with Tri-methyl-H3(Lys9) antibody or rabbit IgG to analyse NF-κB binding sites within *Il6* promoter. **B** Same as in **A,** but with anti-myc antibody (HOPS) or rabbit IgG. Values are the mean ±SD (n=3). Statistically significant changes are shown.

**Supplementary Fig.S5. HOPS immunolocalization in LPS treated RAW 264.7 cells**. Cells were treated with LPS and HOPS localization was observed using the specific antibody labelled in red. Nuclei were DAPI stained (blue). Representative images are shown. Bars, 10 μm.

**Supplementary Fig.S6. Analysis of p65-IkBα endogenous binding**. RAW 264.7 cells were transfected with HOPS and lysates were immunoprecipitated with the specific anti-p65 antibody. Coimmunoprecipitated proteins were revealed with anti-IκBα and anti-p65 antibodies. Total cell lysate input was revealed with anti-p65, anti-IκBα antibodies, and HOPS transfection was probed by anti-HOPS antibody.

**Supplementary Fig.S7. HOPS acts in LPS-mediated IKKγ-activation**. HEK293T cells were cotransfected with Flag-TRAF6 and HA-(mono)ubiquitin in the presence of HOPS or not. Lysates LPS treated (10μg/ml) or untreated cells were IKKγ-immunoprecipitated and immunoblotted with anti-HA to reveal the ubiquitin-bound IKKγ. Total cell lysate input was analysed with the indicated antibodies.

**Supplementary Fig.S8. Analysis of TRAF6/HOPS endogenous binding.** RAW264.7 cells were immunoprecipitated with the specific HOPS antibody and IgG were used as negative control. Co-immunoprecipitated proteins and total cell lysate input were revealed by WB analysis with anti-TRAF6 and anti-HOPS antibodies.
